# Comparison of ferric Carboxymaltose and iron sucrose complex for treatment of iron deficiency anemia in pregnancy- randomised controlled trial

**DOI:** 10.1186/s12884-019-2200-3

**Published:** 2019-02-04

**Authors:** Ambily Jose, Reeta Mahey, Jai Bhagwan Sharma, Neerja Bhatla, Renu Saxena, Mani Kalaivani, Alka Kriplani

**Affiliations:** 1 Department of Obstetrics and Gynecology, All India Institute of Medical Sciences, New Delhi, India; 2 Department of Hematology, All India Institute of Medical Sciences, New Delhi, India; 3 Department of Biostatistics, All India Institute of Medical Sciences, New Delhi, India

**Keywords:** Iron deficiency anemia, Pregnancy, Ferric carboxymaltose, Iron sucrose

## Abstract

**Background:**

To evaluate the efficacy and safety of intravenous Ferric Carboxymaltose. (FCM) in comparison with intravenous Iron sucrose complex (ISC) for treatment of iron deficiency anemia in pregnancy.

**Methods:**

A randomized clinical trial was conducted from (January 2016–August 2017). at a tertiary hospital. Pregnant women diagnosed with moderate to severe iron deficiency anaemia were screened for the study. One hundred patients were randomized to receive either intravenous FCM or ISC. Primary outcome was rise in hemoglobin (Hb) from baseline after 12 weeks. Secondary outcomes were change in RBC indices, serum iron studies, improvement in fatigue scores, number of visits and perinatal outcome.

**Results:**

Mean rise in Hb at 12 weeks was significantly higher in FCM group (29 g/L vs 22 g/L; *p* value < 0.01). FCM was associated with greater improvement in fatigue scores. Number of visits were significantly less in FCM group. No serious adverse events were noted in either group.

**Conclusion:**

Treatment with FCM resulted in rapid replenishment of iron stores in pregnant women with significantly higher Hb rise over a 12 week period. The convenient dosing with lesser number of total doses to complete the treatment will lead to better compliance in community setting.

**Clinical trial registration (www.ctri.nic.in):**

CTRI/2015/09/006224. Registered on 21/07/2017 (Trial registered retrospectively).

## Background

Anemia is one of the major health issues worldwide. Iron deficiency anemia is the most common type of nutritional deficiency affecting both developed and developing countries. An estimate by WHO attributes about 591,000 perinatal deaths and 115,000 maternal deaths globally to iron deficiency anemia directly or indirectly [1]. Prevalence of anemia in South Asian countries is the highest in the world. About half of the global maternal deaths due to anemia occur in South Asian countries and India contributes to about 80% of it [2]. Anemia affects all age groups starting from puberty and adolescence to perimenopausal age. The reasons for high incidence of anemia in India include low dietary intake of iron, poor bio-availability of iron, phytate-rich Indian diet, faulty food habits, chronic blood loss during menses and high prevalence of infections like malaria and hookworm infestations [3]. The condition gets aggravated in pregnancy due to increased demand of the growing fetus.

Prophylactic oral iron is recommended during pregnancy to meet the increased requirement during the antenatal period. The main issue with oral iron therapy is compliance due to associated gastrointestinal side effects like bloating, diarrhea, heartburn, nausea, constipation, and dark stools.

Also, oral therapy is not sufficient for treatment of moderate to severe anemia, especially in the late second and third trimester. Parenteral therapy promises a better response in these patients and can obviate the need for blood transfusions in the antenatal and postpartum period [4]. The most commonly used iron preparation for anemia in pregnancy is iron sucrose complex (ISC). It has negligible safety issues and no test dose is required. The only disadvantage with iron sucrose is limited dose per sitting. The maximum permissible dose is 300 mg per sitting or 600 mg per week. This adds to the total cost of therapy as it requires multiple visits.

The latest addition in i.v. iron preparations is Ferric Carboxymaltose (FCM), which is a dextran free type I iron complex. A lot of studies have been published on the use of FCM for treatment of anemia in the postpartum period and other diseases with associated anemia. But, there is limited literature on the use of FCM in pregnancy. There are very few prospective studies on FCM in pregnancy and no randomized controlled studies comparing FCM and Iron sucrose complex in pregnancy. The present study was conducted to evaluate the efficacy, safety, cost effectivity of FCM compared with ISC for treatment of moderate to severe iron deficiency anemia in pregnancy.

## Methods

The study was conducted as an open-label randomized clinical trial in the antenatal clinic of a tertiary hospital in New Delhi, India from January 2016 to August 2017. Ethical clearance was obtained from institute’s ethics committee and the trial was registered with Clinical Trial Registry of India (CTRI) CTRI/2015/09/006224.Pregnant women attending the antenatal clinic between 16 and 36 weeks period of gestation were screened for the study. Patients with Hb > 60 g/L and < 100 g/L and iron deficiency anemia (IDA) were enrolled for the study. Informed written consent was taken from all the patients before recruitment into the study. Exclusion criteria were: anemia due to causes other than IDA; any chronic infections like hepatitis and HIV; serum transaminases more than 1.5 times the upper limit of normal; serum creatinine level of more than 2.0 mg/dL or history of allergic reaction to intravenous iron infusion. One hundred pregnant women were randomized using a computer-generated block randomization table into two groups in a 1:1 ratio and were administered either FCM or ISC.

On enrollment, a detailed clinical history (menstrual, obstetric), previous treatment history including iron therapy, compliance with oral iron and chronic medical illness was taken. Detailed examination including anthropometry, general physical examination and obstetric examination was done. Routine antenatal investigations were done according to the standard departmental protocol. Investigations specific to anemia included hemogram, reticulocyte count and peripheral blood smear, red cell indices including mean corpuscular volume (MCV), mean corpuscular hemoglobin (MCH), mean corpuscular hemoglobin concentration (MCHC), red cell distribution width (RDW), Hb electrophoresis, serum ferritin levels, serum iron, total iron binding capacity (TIBC) and transferrin saturation were done. Fatigue measurement was done by a Linear Analogue Scale Assessment (LASA) that recorded scores between 0 (no fatigue) and 10 (worst possible fatigue).

### Iron requirement was calculated according to Ganzoni’s formula [5]

$$ \mathsf{Iron}\ \mathsf{requirement}\ \left(\mathsf{mg}\right)=\mathsf{Total}\ \mathsf{iron}\ \mathsf{deficit}\ \left[\mathsf{mg}\right]=\mathsf{BW}\ \left[\mathsf{kg}\right]\ \mathsf{x}\ \left(\mathsf{target}\ \mathsf{Hb}\left(\mathsf{14}\;\mathsf{g}/\mathsf{dL}\right)-\mathsf{actual}\ \mathsf{Hb}\right)\ \left[\mathsf{g}/\mathsf{dL}\right]\times \kern0.37em \mathsf{0.24}+\mathsf{storage}\ \mathsf{iron}\ \left(\mathsf{1000}\right)\ \left[\mathsf{mg}\right] $$

After calculating total iron deficit, patients in the FCM group were administered i.v. FCM (InjOrofer FCM, Emcure Pharmaceuticals Ltd., Pune, India). Maximal dose per sitting was 1000 mg which was diluted in 200 ml 0.9% normal saline and administered as an IV infusion over 30 min (Due to the limited availability of safety data for its use in pregnancy, a longer infusion protocol (30 min) than recommended by the manufacturer (15 min) was used). Subsequent doses (if needed) were planned on day 7 and day 14 and doses were rounded off to the nearest 100 mg. Patients in ISC group were administered IV ISC as 300 mg(Inj Orofer S, Emcure Pharmaceuticals Ltd., Pune, India) in 200 ml NS over 15-20 min twice weekly till dosage was completed, not to exceed 600 mg per week. The general condition of the patient, blood pressure and pulse rate were noted before infusion and every five minutes during infusion and fetal heart rate monitoring was done before and after infusion.

All women were administered antihelminthic therapy with tablet mebendazole 100 mg twice daily for three days and given 5 mg Folic acid once daily. Any minor or major adverse effects were noted. All patients were followed up after 3, 6 and 12 weeks of initiation of treatment. Hb, RBC indices and serum iron studies were done at each visit. Patients reported minor or major adverse events at follow-up visits. Primary outcome was change in Hb level from baseline after 12 weeks. Secondary outcomes were change in ferritin levels, improvement in serum iron studies and RBC indices, RDW, change in fatigue levels, safety and side effects of treatment and perinatal outcome.

From an earlier study by Christoph et al. (2012) where the final Hb after administering iron sucrose Hb increased from 95.6 g/L to 110.4 g/L with a standard deviation of 11.9, taking non-inferiority limit difference in mean of Hb between the two groups as 10 g/L, and the expected mean difference as zero, and standard deviation as 11.9, alpha error 5% and power of the study as 90%, the estimated sample size was 24 per group. Considering 10% loss to follow up, a minimum of 27 iron-deficient pregnant anemic women needed to be included in each group in the study; hence 50 patients were included in each group.

Data were presented as number (%) or mean ± SD/median (min-max) as appropriate. Baseline categorical variables were compared between the groups using Chi-square/Fisher’s exact test and continuous variables were compared using Student’s t-test/Wilcoxon Rank Sum test.

Analysis was done according to intention-to-treat analysis. We considered non-inferiority margin of 10 g/L, which means that when the upper limit of 95% CI for the estimated difference in Hb after administering FCM vs ISC exceeds 10 g/L, FCM is inferior to ISC. Confidence interval for a difference between two means was used to calculate 95% CI for Hb (g/L). The other study outcomes, namely MCV, MCH, MCHC, RDW, reticulocyte count, serum ferritin, serum iron, TIBC, transferrin saturation, which were measured over a period of time was analysed and compared using generalized estimating equation. The variables that do not follow normal distribution were compared between the groups using Wilcoxon Rank Sum test. The *p* value less than 0.05 was considered statistically significant.

All statistical analysis was carried out using Stata 12.0 (StataCorp LP, College station, Texas, USA).

## Results

**Fig. 1:**
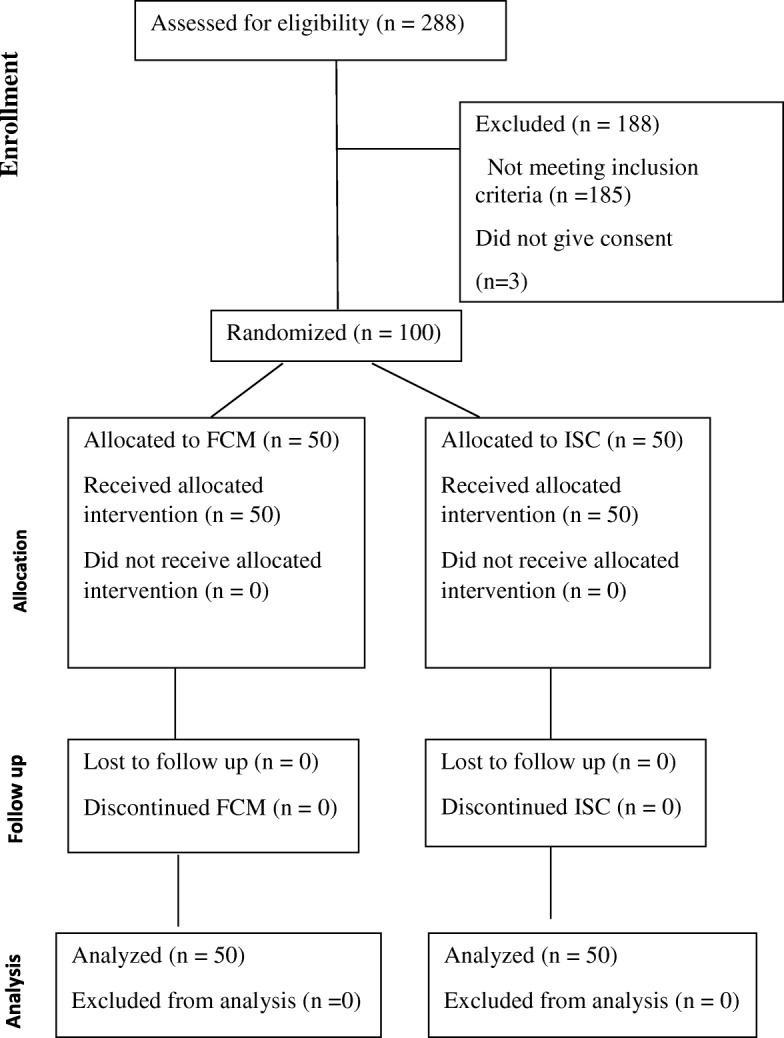
Consort diagram

According to inclusion and exclusion criteria, 100 pregnant women were found eligible, gave consent and were randomised into two groups of 50 each. All 100 patients completed the treatment and were included in analysis (Fig. 1).

Table 1 shows the demographic characteristics of the study population which were comparable in two groups.

**Table 1:** Baseline characteristics of study participants

Characteristics	FCM group (*n* = 50)	ISC group (*n* = 50)	*P* value
Age (years)	27.5 ± 3.9	26.2 ± 3.6	0.10
Weight (kg)	57.3 ± 4.8	57.4 ± 5.8	0.97
BMI (kg/m^2^)	20.5 ± 1.5	20.5 ± 1.8	0.95
Type of anemia			
Moderate (70-99 g/L)	49	48	
Severe (< 70 g/L)	1	2	
Baseline Hb(g/L)	85.7 ± 8.9	86.7 ± 8.6	0.57
MCV (fl)	75.5 ± 6.0	75.3 ± 5.3	0.82
MCH (pg)	24.4 ± 2.7	24.4 ± 2.2	0.94
MCHC (g/dL)	29.7 ± 1.9	30.0 ± 1.8	0.41
RDW (%)	21.9 ± 6.0	20.5 ± 5.5	0.24
Reticulocyte count	1.0 (0.5–3.2)	1.1 (0.5–6.1)	0.49
S. Iron (μg/dL)	28.5 (14–78)	32 (14–74.1)	0.15
S. Ferritin (μg/L)	7.9 (0.4–22.3)	9 (0.94–23)	0.46
TIBC (μg/dL)	493.8 ± 106.5	530.0 ± 96.0	0.07
Transferrin saturation (%)	8 (0.4–30.5)	12.5 (0.03–19.1)	0.035
Period of gestation at first dose (weeks)	27.5 ± 4.6	26.4 ± 4.7	0.26

The mean calculated iron requirement was comparable in the FCM and ISC group (1739.6 ± 105.5 vs 1730.4 ± 121.9 mg; *p* = 0.69), however, the number of doses required to build up the deficit was significantly less in FCM group compared to ISC group (2.0 ± 0 vs 6.04 ± 0.45; *p* value < 0.001). Therefore, the time required to administer the total drug dose was 1 week in FCM group (2 doses on day 0 and 7), while in ISC group was 3 weeks (2 doses per week).

Primary outcome was rise in Hb from baseline at the end of 12 weeks (Table 2). The mean rise in Hb at 12 weeks was significantly higher in FCM group than ISC group (29 g/L vs 22 g/L; p value < 0.001 (Table 2).

**Table 2:** Rise in hemoglobin at 12 weeks from baseline

Hb (g/L)	FCM group (*n* = 50)	ISC group (*n* = 50)	Difference (95% CI)
Baseline	85.7 ± 8.9	86.7 ± 8.6	−1(−4.49, 2.49)
Endline (12 weeks)	115.3 ± 4.6	108.8 ± 4.4	6.5 (4.7, 8.29)
Change in Hb	29.6 ± 8.2	22.1 ± 8.2	−7.5 (−4.24, −10.76)*

Data presented as mean ± SD; *statistically significant and 95% CI calculated using non-inferiority margin of 10 g/L of change in Hb at 12 weeks from baseline

**Fig. 2:**
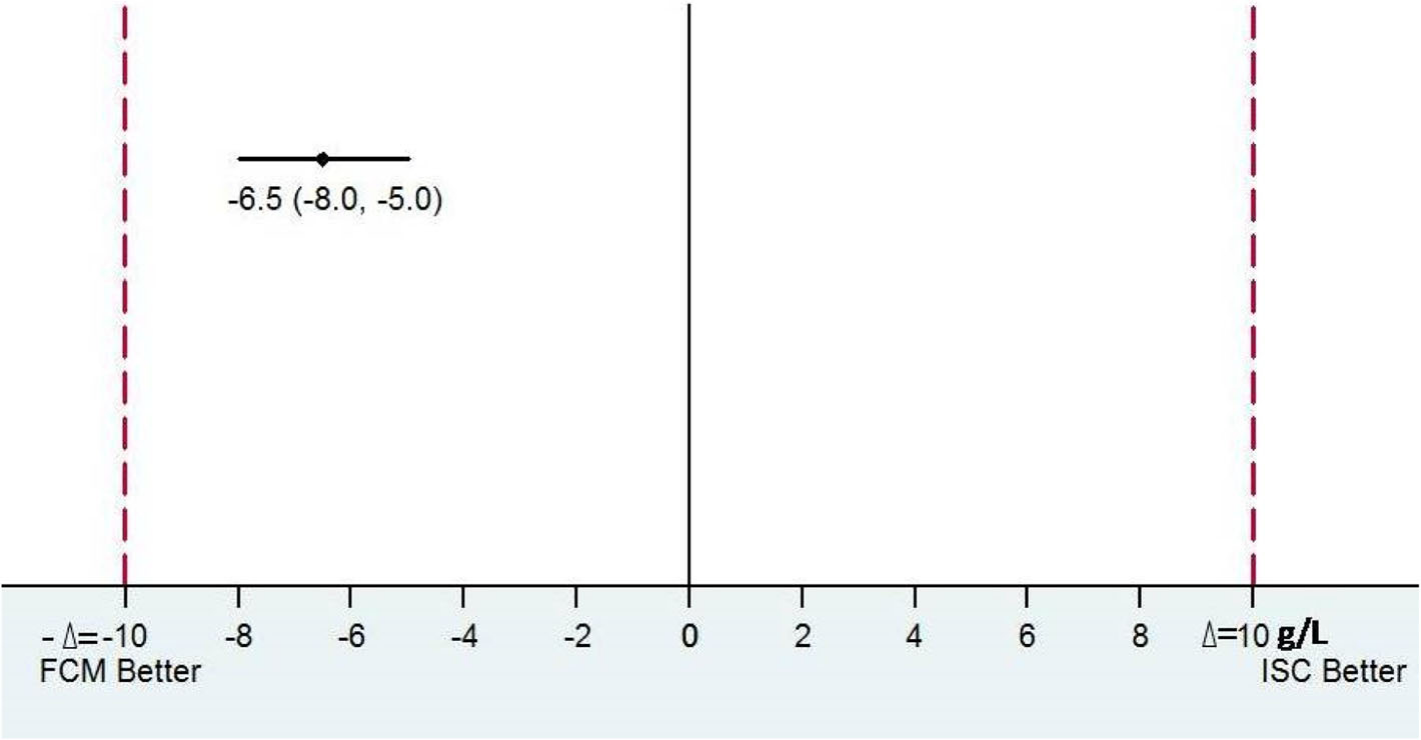
Non-inferiority of FCM against ISC in change of Hb at 12 weeks from baseline (non-inferiority of 10 g/L)

FCM was non-inferior to ISC in causing change of Hb at 12 weeks from baseline, taking a non-inferiority margin of 10 g/L (Fig. 2).

Tables 3 and 4 show hematological parameters and serum iron studies respectively at baseline, 3 weeks, 6 weeks and 12 weeks after the initiation of therapy.

**Table 3:** Hematological parameters at different time points in the study

Parameter	FCM (*n* = 50)	ISC (*n* = 50)	Differences in mean between study groups (95% confidence interval)	*P* value
Hemoglobin(g/L)				
Baseline	85.7 ± 8.9	86.7 ± 8.6	1(−4.49,2.49)	0.57
3 weeks	10 6 ± 12.1	100.1 ± 7.2	6(2.0, 9.8)	0.003
6 weeks	114.8 ± 6.9	108.2 ± 6.8	7(3.9,9.3)	< 0.001
12 weeks	115.3 ± 4.6	108.8 ± 4.4	6.5 (4.7,8.29)	< 0.001
Mean corpuscular volume (fL)				
Baseline	75.5 ± 6.0	75.3 ± 5.3	0.2 (−1.93,2.46)	0.82
3 weeks	82.2 ± 5.5	81.4 ± 4.4	0.8 (− 1.20, 2.70)	0.45
6 weeks	86.4 ± 3.4	86.2 ± 4.2	0.2 (− 1.26, 1.73)	0.76
12 weeks	88.9 ± 2.1	88.2 ± 2.4	0.7 (− 0.25, 1.50)	0.16
Mean corpuscular hemoglobin (pg)				
Baseline	24.4 ± 2.7	24.4 ± 2.2	−0.03 (− 0.99, 0.93)	0.94
3 weeks	27.5 ± 1.6	27.4 ± 1.7	0.07 (− 0.56, 0.71)	0.82
6 weeks	29.0 ± 1.4	28.8 ± 1.3	0.24 (− 0.28, 0.76)	0.37
12 weeks	30.4 ± 1.4	29.8 ± 1.4	0.6 (0.001, 1.1)	0.049
Mean corpuscular hemoglobin concentration (g/dL)				
Baseline	29.7 ± 1.9	30.0 ± 1.8	−0.30 (−1.005, 0.41)	0.41
3 weeks	31.7 ± 1.8	31.8 ± 1.5	− 0.05 (− 0.69,0.59)	0.88
6 weeks	32.9 ± 1.5	33.1 ± 1.1	−0.26 (− 0.78, 0.25)	0.31
12 weeks	33.7 ± 0.9	33.7 ± 0.9	−0.002 (− 0.36, 0.35)	0.99
Red cell distribution width (%)				
Baseline	21.9 ± 6.04	20.5 ± 5.49	1.4 (−0.89, 3.61)	0.24
3 weeks	20.7 ± 5.34	20.0 ± 4.09	0.7 (− 1.17, 2.54)	0.47
6 weeks	16.2 ± 3.19	15.8 ± 2.89	0.4 (− 0.85, 1.53)	0.58
12 weeks	13.6 ± 1.51	13.5 ± 1.38	0.1 (− 0.45, 0.68)	0.68

Data presented as mean ± SD

**Table 4:** Serum iron studies at different time points in the study

S.Iron (μg/dL)			
Baseline	28.5 (14–78)	32 (14–74.1)	0.15
3 weeks	178 (49.7–345)	187 (40–628.9)	0.30
6 weeks	156 (72–250)	152 (71.6–561.6)	0.78
12 weeks	112 (72–187)	107.5 (65–327)	0.60
S. Ferritin (μg/L)			
Baseline	7.9 (0.4–22.3)	9 (0.94–23)	0.46
3 weeks	343 (25.7–843)	298 (13–771)	0.02
6 weeks	291.5 (123–643)	233.3 (54.5–645)	0.44
12 weeks	187.5 (69–443)	145.5 (40–382)	0.13
Transferrin saturation (%)			
Baseline	8 (0.4–30.5)	12.5 (0.03–19.1)	0.035
3 weeks	35.85 (12.2–64)	39.81 (14–138.4)	0.50
6 weeks	37.65 (13.6–53)	37 (16.82–84.5)	0.84
12 weeks	32.7 (5–46)	34.5 (19.6–65.7)	0.53
TIBC (μg/dL)			
Baseline	493.8 ± 106.5	530.0 ± 96.0	0.07
3 weeks	413.4 ± 76.9	439.9 ± 86.0	0.10
6 weeks	348.4 ± 72.4	354.9 ± 88.4	0.68
12 weeks	284.7 ± 50.9	290.2 ± 60.7	0.62

Data expressed as mean ± SD/median (min-max)

**Fig. 3:**
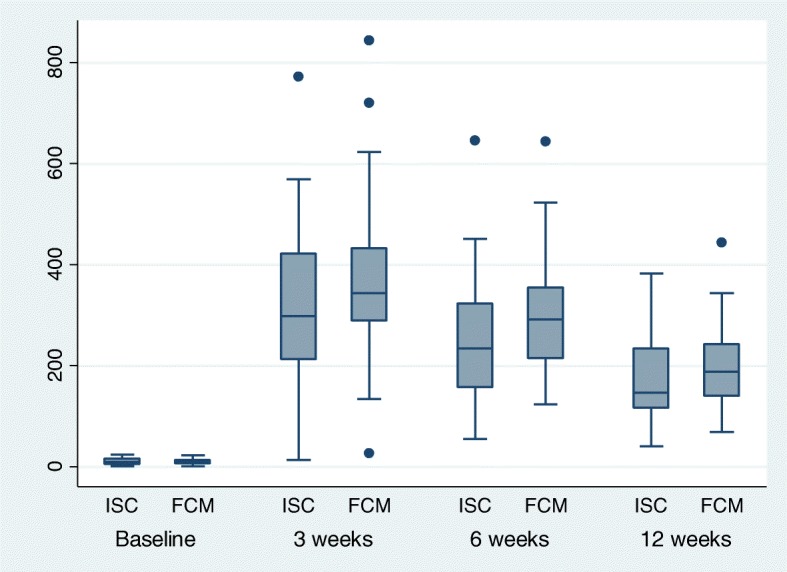
Box and whisker plot of serum ferritin levels in patients in FCM and ISC groups over 12 weeks

As shown in Table 3, Hb started rising at a higher rate at 3 weeks interval which persisted till the end of study at 12 weeks. The baseline serum ferritin in FCM and ISC group was 7.9 (0.4–22.3) μg/L and 9 (0.94–23) μg/L respectively. Serum ferritin level was significantly higher in FCM group compared to ISC at 3 weeks but this difference disappeared at end of 12 weeks showing comparable levels between the two groups (Fig. 3).

Though there was no significant difference in baseline, (*p* value = 0.568), the LASA scores were significantly lower in FCM group compared to ISC group at 12 weeks (*p* value = 0.048).

All the patients in both groups received complete calculated dose of drug. No serious adverse events were noted in either group. There were no cancellations of injections due to minor or major adverse reactions. There was one instance of injection site reaction in FCM group and two instances in ISC group. Two patients in ISC group had mild epigastric pain after injection within an hour of injection in ISC group, but this was transient mild not associated with nausea or vomiting and managed conservatively. One patient in FCM group was found to have serum transaminases more than double the baseline value at 3 weeks, but it normalized at 6 weeks. Hypophosphatemia was found in 2 (4%) and 3 (6%) patients in FCM and ISC group respectively, but all had normal serum phosphate values at 12 weeks.

Seven (14%) patients in FCM group had a preterm delivery before 37 completed weeks of gestation, of which only one was within 2 weeks of last dose. Six (12%) women in ISC group had a preterm delivery, while one patient who received ISC from 17 weeks period of gestation had a spontaneous abortion at 22 weeks and 5 days period of gestation. The birth weight in FCM group was 2834.1 g and in ISC group was 2864.7 g; the difference was not statistically significant (*p* value = 0.73).

The cost of total therapy was INR 6872.4 ± 379.7 and INR 6566.3 ± 449.8 respectively (p value = 0.0004) in FCM group and ISC group. But this just included the cost of the drug and the cost of consumables (i.v. cannula, i.v. drip set, and normal saline).

## Discussion

The present study compared two intravenous iron preparations for the correction of iron deficiency anemia in pregnancy. FCM was found non-inferior to ISC in correction of anemia and it led to significantly higher and rapid Hb rise as compared to ISC group and with significantly less number of visits.

Iron sucrose has been the standard of care for parenteral iron therapy for treatment of anemia in pregnancy. However, the main disadvantage with iron sucrose is limited maximum permissible dose per week thus need of multiple visits to deliver the required iron dose, while FCM can be administered in a larger amount at a time.

The first study on the use of FCM for treatment of IDA in pregnancy was published by Christoph P et al. [6]. The study concluded comparable safety and tolerability of FCM to ISC and that FCM offers the advantage of a much higher iron dosage at a time reducing the need for repeated applications and increasing patients’ comfort. The authors documented a comparable rise in Hb levels at the end of the study. The current study, in contrast, showed significantly higher Hb levels in FCM group as compared to ISC group after 12 weeks.

Breymann C et al. compared FCM with oral iron therapy for treatment of iron deficiency anemia in pregnancy. Hb levels improved at comparable rates in both groups. Patients in FCM group had significantly more women who achieved Hb > 110 g/L and within a shorter time frame. The authors concluded to consider FCM to be first-line treatment option for correction of IDA especially in the third trimester of pregnancy [7].

Body iron stores are largely determined by serum ferritin levels. Froessler et al. have documented significantly increased ferritin levels after FCM infusion in patients with anemia and in women with iron deficiency and no anemia [8, 9]. In the present study, serum ferritin levels were comparable in two groups at baseline and at the end of study after 12 weeks. It can be inferred that though FCM causes a rapid rise in iron stores, over a long term ISC is equally able to give comparable supplementation for replenishment of iron stores.

Fatigue and poor quality of life are known symptoms of anemia that can be improved by treatment. The present study used a LASA to measure improvements in fatigue scores and a 10 point numeric scale to assess the feeling of well-being. LASA scores were significantly lower in FCM group compared to ISC group at 12 weeks. This was in agreement to two studies by Van Wyck et al. [10, 11], in non-pregnant females, where FCM was demonstrated to significantly improve patient quality of life scores and fatigue levels when compared to ISC.

In a recent systematic review to compare different injectable iron preparations in pregnancy, the authors failed to document the safety of any of the injectable iron therapies over others. The choice of injectable iron therapy is mainly determined by cost and convenience of administration [12].

Previously published studies have compared the cost of therapy in FCM and ISC treatment and have documented significantly less cost in FCM group [13, 14].In the study, the drug was provided free of cost to the patients under the JSSK (Janani Shishu Suraksha Karyakram) scheme for pregnant women by the Indian Government. The total cost of drug was analyzed in both the groups in the present study and showed significantly higher cost in FCM group (difference INR 306.1 (140.93, 471.3). But this analysis did not include travel costs and the number of working days lost due to travel which would have been more in ISC group as the number of visits was significantly higher in ISC group. The overall cost of therapy would have been higher due to the need of multiple visits required to receive the complete dosage in ISC group.

In addition to lesser number of visits required for completion of therapy, present study showed early and persistently higher rise in Hb in FCM group from 3 weeks onwards which continued till 12 weeks. Ferritin level though higher at 3 weeks in FCM group, the difference disappeared at 12 weeks. The finding indicated that both FCM and ISC are similar in replenishment of stores and rise in Hb is more and earlier with FCM. This is important in pregnant women presenting with moderate to severe anemia especially in third trimester.

## Conclusion

FCM is a safe intravenous agent in pregnancy and is non-inferior to the current standard therapy (iron sucrose complex) for the treatment of iron deficiency anemia in pregnancy. FCM has the advantage of a large dose administration per sitting, early rise in Hb level, lesser total number of required doses (convenient dosing), hence lesser number of hospital visits and total cost involved in transportation, equipment required for infusion and the discomfort caused to the patient due to multiple needle pricks. Improvement in fatigue scores were better with FCM over 12 weeks. Significantly shorter duration of treatment when considered in a community setting with the patient friendly dosing, may translate to better patient compliance to treatment.

## Abbreviations

FCM: Ferric carboxymaltose
IDA: Iron deficiency anaemia
ISC: Iron sucrose complex
LASA: Linear analogue scale
MCH: Mean corpuscular hemoglobin
MCHC: Mean corpuscular hemoglobin concentration
MCV: Mean corpuscular volume
